# MetaboClust: Using interactive time-series cluster analysis to relate metabolomic data with perturbed pathways

**DOI:** 10.1371/journal.pone.0205968

**Published:** 2018-10-29

**Authors:** Martin J. Rusilowicz, Michael Dickinson, Adrian J. Charlton, Simon O’Keefe, Julie Wilson

**Affiliations:** 1 Department of Computer Science, University of York, York, United Kingdom; 2 York Centre for Complex Systems Analysis, University of York, York, United Kingdom; 3 Fera Science Ltd, York, United Kingdom; 4 Department of Mathematics, University of York, York, United Kingdom; 5 Department of Chemistry, University of York, York, United Kingdom; University of Tsukuba, JAPAN

## Abstract

**Motivation:**

Modern analytical techniques such as LC-MS, GC-MS and NMR are increasingly being used to study the underlying dynamics of biological systems by tracking changes in metabolite levels over time. Such techniques are capable of providing information on large numbers of metabolites simultaneously, a feature that is exploited in non-targeted studies. However, since the dynamics of specific metabolites are unlikely to be known *a priori* this presents an initial subjective challenge as to where the focus of the investigation should be. Whilst a number of feed-forward software tools are available for manipulation of metabolomic data, no tool centralizes on clustering and focus is typically directed by a workflow that is chosen in advance.

**Results:**

We present an interactive approach to time-course analyses and a complementary implementation in a software package, MetaboClust. This is presented through the analysis of two LC-MS time-course case studies on plants (*Medicago truncatula* and *Alopecurus myosuroides*). We demonstrate a dynamic, user-centric workflow to clustering with intrinsic visual feedback at all stages of analysis. The software is used to apply data correction, generate the time-profiles, perform exploratory statistical analysis and assign tentative metabolite identifications. Clustering is used to group metabolites in an unbiased manner, allowing pathway analysis to score metabolic pathways, based on their overlap with clusters showing interesting trends.

## Introduction

While the dynamic nature of the metabolome has been acknowledged for some time [1], it is the ever increasing throughput abilities of technologies such as LC-MS and NMR that have permitted the inspection of metabolites over meaningful timescales. As a series of fixed frame snapshots, time-series analyses allow processes to be monitored over time and permit a more complete view of the underlying mechanisms to be drawn. Time course metabolomic studies have been employed for a wide variety of purposes, including nutrition [2], temperature response [3], abiotic [4,5] and biotic [6] stress in plants, renal function [7], toxicity [8], circadian rhythms [9] [10], cancer treatment [11] and drug reactions [11]. There is a wealth of information available about metabolomic and proteomic responses, with databases spanning the full–omics hierarchy: METLIN (metlin.scripps.edu), HMDB (http://www.hmdb.ca), NCBI (www.ncbi.nlm.nih.gov), EMBL-EBI (www.ebi.ac.uk), KEGG (www.genome.jp/kegg) and MetaCyc/BioCyc (metacyc.org, biocyc.org) to name a few. From these data we are able to provide links between individual metabolites, pathways, proteins and genes.

Given a time-series, univariate tests such as ANOVA (analysis of variance) and t-tests provide simple, easy to use methods of quantifying the significance of system change. However, they suffer from several problems when applied to the analysis of large metabolomic datasets, as typically acquired through non-targeted LC-MS, GC-MS or NMR. Most notably, the likelihood of making type 1 errors due to repeated tests increases with the number of variables, which is typically large for untargeted studies. Correction methods such as the Bonferroni correction [12] can reduce the chances of such errors, however these corrections mandate certain assumptions and can lead to an increase of type 2 errors [13]. Even with correction, as large numbers of metabolites are often present, the results can be difficult to interpret. Another issue with analyses such as the t-test is that they require selection of “before” and “after” time-points, who’s range cannot be known in advance without data dredging. Given the expenditure of data collection, it is important to make use of the full time-series rather than focus on specific subsets. Alternative analyses such as Pearson correlation (univariate) or Partial Least Squares Regression (PLSR, multivariate), can be used to identify the variables showing correlation with time and therefore can extract certain time-specific information from the data. However, features showing trends of potential interest that do not correlate linearly with the specified function of time will remain undetected. For example, quadratic trends are not detected via a direct Pearson correlation with time. Such trends can however be identified via correlation analyses using suitable time-course “templates”, sourced, for instance, from a known metabolite of interest, however, this again requires information to be known in advance.

More advanced techniques for identifying differential profiles are commonly used in time course gene expression studies. For instance, maSigPro uses a two stage method to filter genes based on their expression profile [14], BATS uses a Bayesian approach to rank genes of interest [15], which can offer increased accuracy for time courses with more data points [16], and the EDGE software can identify time dependent changes [17]. While these methods can rapidly identify differences between experimental groups their univariate nature provides a specific, rather than comprehensive, view of the data [18].

Rather than selecting interesting time-series manually, clustering algorithms are able to naturally group together sets of related trends in an unbiased manner. A number of different clustering methods have been employed in the analysis of biological time series data to date, including hierarchical cluster analysis (HCA) [19], self-organizing maps (SOMs) [20] and Bayesian clustering [21].

Despite the array of techniques available, the “no free lunch” theorem [22] suggests that it is unlikely that any one technique is suited to the exploration of any and all datasets, and a number of software tools have been developed that permit exploratory analysis of metabolomic data. Existing software tools fall into three categories; script based (e.g. XCMS [23]), web based GUIs (e.g. XCMS-Online [24], MetaboAnalyst [25]) and client-based GUIs (e.g. MZmine [26]). While some focus on particular tasks (e.g. MetAlign [27]), others target the complete set of possible manipulations (e.g. XCMS-Online). Few tools visualize the clustering of metabolomic data (MZmine, MetaboAnalyst, XCMS-online), and clustering is usually seen as an output rather than as the target for further exploration. Furthermore, of these tools, only MZmine offers rapid data exploration through a rich client application. With the exception of hand crafted R-scripts using XCMS, no GUI offers a dynamic workflow.

**Fig 1:**
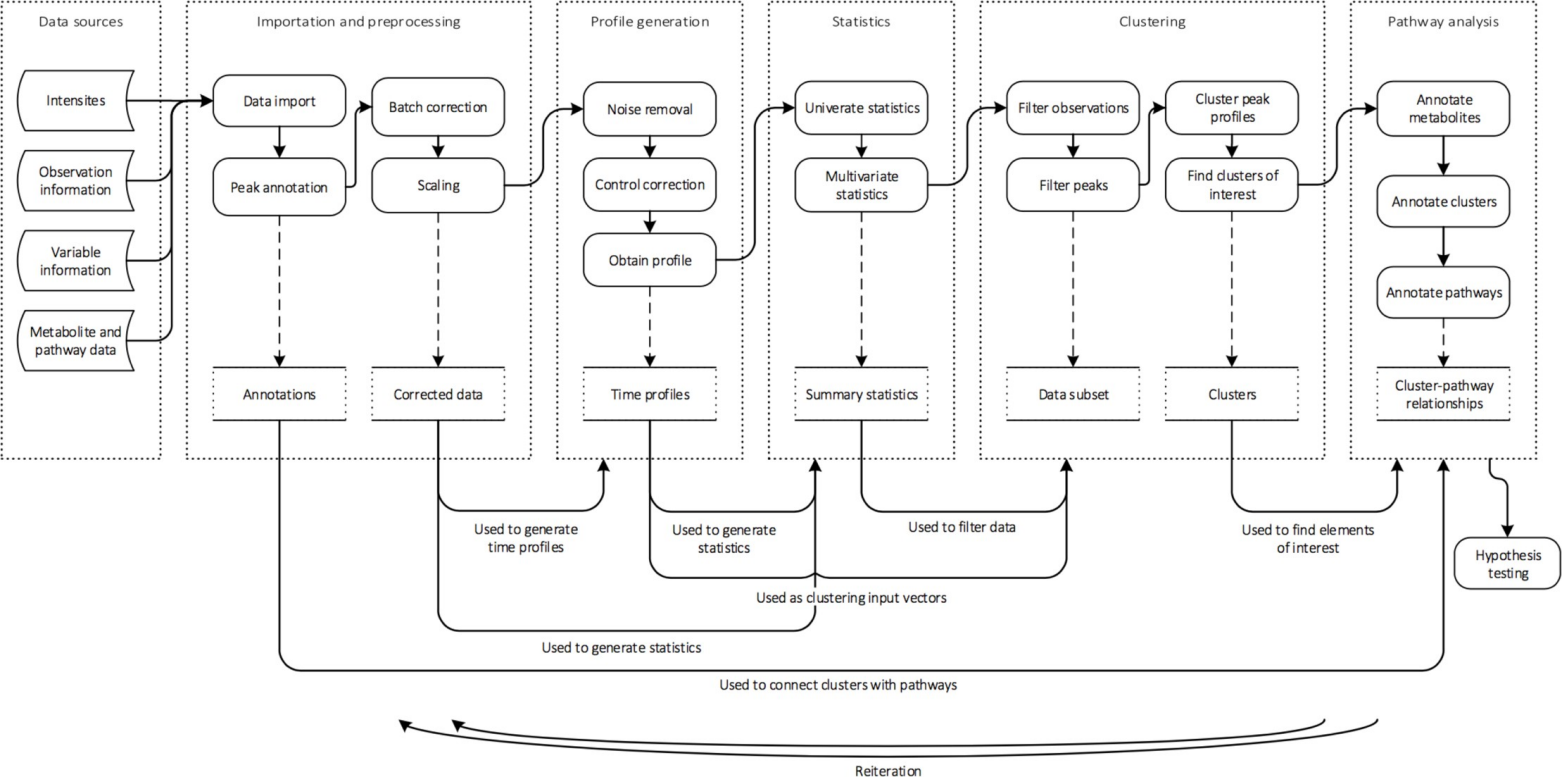
Flow chart showing the various analysis stages in MetaboClust and how they can be connected. It is possible to re-run the analysis from various stages, but, for simplicity, the complete set of reverse arrows has not been shown.

Here we present a graphically interactive workflow for metabolomic time series analysis in the form of a software package, MetaboClust. We account for the fact that in large, untargeted studies the workflow is unlikely to be known at the outset and therefore place focus on fast, highly interactive visualizations, which allow the user to navigate quickly between clusters, features, metabolites and pathways. Taking the model shown in S1 Fig, we developed a dynamic workflow, as shown in Fig 1, which allows the user to preview the effects of potential data set manipulations at stages further down their pipeline.

To demonstrate use of the software, we present two case studies on plant stress; the model legume, *Medicago truncatula* and the weed species, *Alopecurus myosuroides*. Using clustering and online databases we draw links between groups of metabolites showing similar pathway perturbations, and pathways sourced from the BioCyc database.

### Workflow

#### Data import

The *data sources* comprise the table of peak intensities, observation and peak information, and metabolite and pathway data. Peak intensities are represented as a CSV file organized with one observation per row and one variable per column, produced by peak-pickling in software such as XCMS or Progenesis QI. The meta-data on the observations (rows) and variables (columns) is provided as experimental and peak information. Experimental information includes experimental group and time as well as technical information, such as batch and acquisition order, whereas peak information depends on the data collection technique, but provides details that allow the peak to be identified, for example *m/z* and retention time or NMR shift. Metabolite data can be imported as a list of metabolites likely to be represented by the peaks in the data, with information such as known accurate mass and potential adducts and pathway data, for example from MetaCyc/BioCyc databases, can also be imported.

#### Batch correction and scaling

LC-MS spectra often exhibit both differences in reported intensity between analytical batches, as well as within-batch drift. Batch correction involves the use of methods to account for these differences and brings the recorded sample intensities of all observations into line with respect to each other. Batch correction techniques follow the general formula:

$$X_{p,b,i}^{\prime }=X_{p,b,i}\frac{R_{p}}{C_{p,b,i}}$$

or, perhaps more intuitively:

$$X_{p,b,i}^{\prime }=X_{p,b,i}-C_{p,b,i}+R_{p}$$

where *X*_*p*,*b*,*i*_ is the intensity of peak *p* for sample *i* within batch *b*, prior to correction and *Xʹp*,*b*,*i* is the corrected value. *C*_*p*,*b*,*i*_ represents the correction factor and *R*_*p*_ represents a rescaling factor, which allows the relative intensity of the peaks to be maintained.

The choice of correction factor (*C*_*p*,*b*,*i*_) is core to the method and allows the intensity for a particular peak (*X*_*p*,*b*,*i*_) to be rescaled to account for variation related to batch (*b*) or acquisition order (*i*). The most basic correction factor is the average of the QC samples for the batch so that *C*_*p*,*b*,*i*_ is the same for all *p* and *i* in a particular batch, *b*. More complex rescaling factors *C*_*p*,*b*,*i*_ can be determined from a smooth trend over *i*, generated from the QC samples for each particular batch. Finally, the rescaling factor R_*p*_ simply returns the peak to a suitable scale and is generally set to the average intensity of the peak, either for the batch or the whole dataset. A simple example to explain batch correction, using correction factors determined by linear regression is given in S2 Fig.

QC based corrections are well understood and widely used, but recent papers have shown that QC-based corrections can exacerbate rather than improve batch differences in cases where the QC samples do not accurately reflect the trend in the experimental samples [28]. “Background-correction” methods offer an alternative method to reduce the differences between biological replicates whilst enhancing the differences between experimental groups. This can be seen visually in principal components analysis (PCA) plots and quantitatively using relative standard deviations between replicates, F-tests to compare the between-group and within-group variances [29] or the Bhattacharrya distance [28]. Both QC and background-correction methods are available in MetaboClust and data visualization allows the user to determine the most appropriate correction method for their data. Full details and validation of the background correction method employed can be found in [29].

Data scaling, performed before or after batch correction, allows all the recorded intensities to be adjusted so that all peaks contribute equally and prevents metabolites with greater intensity from dominating the analysis. A variety of scaling methods are available in MetaboClust, including auto-, pareto, range and vast scaling [30].

#### Noise removal and time-profile generation

Biological or technical replicate observations are frequently used to provide a measure of noise and a means to increase the accuracy of the data. Replicates may be combined to remove the biological or technical variation using a simple average, such as the mean or, as it is less likely to be affected by outliers, the median, to provide a single time course profile for each experimental group. Noise removal can also be achieved by applying a smoothing function to the time profile for any particular peak. The moving average provides a simple smoothing algorithm in which each time-point is assigned the running average (mean or median) of all observations within a pre-specified distance of that time-point (i.e. within a window of designated width).

#### Control correction

Changes in metabolite intensities over time may not necessarily relate to the experimental conditions of interest. In plant studies a number of compounds will be growth related and therefore show predictable trends with age. Other fluctuations with environmental conditions such as light or temperature may also be present. Control correction is applied to account for changes in experimental groups that also exist in the control group, making other changes in the experimental groups easier to interpret. This is achieved by subtracting the time profile obtained for the control group from the data for the experimental groups. The method used to obtain the profile for the control group may or may not be the same as the method used in the time-profile-generation stage.

#### Statistics and filtering

After noise removal and control correction, the resulting time profiles are used to generate statistics and create input vectors for cluster analysis. Standard univariate and multivariate statistical tests, such as t-tests, Pearson Correlation, PCA and partial least squares regression (PLSR) can be used for exploratory analyses of the data. The results of such tests can also be used as a “filter” for further stages of analysis. In particular, when data are scaled, a time profile that is essentially flat could appear erratic and therefore lead to clusters based on spurious data points rather than trends of real interest. Such profiles can be identified and excluded using *t*-tests to compare the data from each of the experimental groups to the control group as follows.

For each experimental group in turn, obtain the value of the *t*-test comparing the data for all time-points and replicates with the control group.

1. Consider the most significant (lowest *p*) of these tests as the final significance value *p*_*min*_
2. If *p*_*min*_ *> α*, where *α* is the chosen confidence limit, exclude the peak from the clustering algorithm and mark as “insignificant”.

Here the *t*-test is only used to detect time-series with little deviation from the base line and no statistical conclusions are drawn from the resulting *p*-values. Experimental observations may also be filtered, for example to remove outliers. The resulting “data subset” is then used as the input into the following cluster analysis stage.

#### Cluster analysis

Cluster analysis allows peaks with similar time profiles to be grouped together. This produces a summary of the types of trends encountered in the data and can also be used for data reduction by filtering peaks to include only those showing interesting patterns. What makes a pattern of interest will vary between experimental considerations and with individual users. MetaboClust offers an advantage over automated selection methods in that the immediate visualization allows the user to make informed decisions on how to proceed, for example on the number of clusters, without a substantial time overhead. While clustering is usually described as operating on “observations”, the objects to be clustered here are not the experimental observations, but the time-course profiles of the individual peaks, which we will refer to as input vectors for clarity. Input vectors may be time profiles for a specific experimental group and peak, or the time profiles for the experimental groups may be concatenated to provide a single input vector for each peak.

A number of clustering algorithms can be used within MetaboClust, for example k-means, HCA or a method, such as DBSCAN (https://cran.r-project.org/web/packages/dbscan/dbscan.pdf implemented via R. Implementation of a deterministic variant of the optimized *k*-means algorithm, *k*-means++ [31], which we call *d-k-*means++, allows rapid visual analysis as various parameter values are explored. The *k*-means++ algorithm operates by providing a more organized structure to the initial cluster centers. Whilst traditional *k*-means starts with a set of random centers, *k*-means++ generates the centers iteratively, using a probability function to determine which observations new centers are created from. Here, we have first replaced the probability function with a parsimony function that always selects the most distant vector. Secondly, we have replaced the initial random generation by the selection of a “seed” observation on the edge of the search space.

1. Select a seed observation on the edge of the search space, such as the most distant observation from the average, or one with a marked profile (e.g. high correlation with time).
2. Create a cluster centered on the seed observation.
3. Compute the squared distances *D*_*i*_^2^ between each observation, *x*_*i*_, and the nearest cluster center.
4. Select the most distant observation from any cluster center, i.e. *m = arg max*_*i*_*(D*_*i*_^*2*^*)*.
5. Create a new cluster center on the selected observation, *x*_*m*_.
6. Repeat 3, 4, 5 until *k* exemplars have been chosen.
7. Lloyd’s algorithm [32] or similar is then used, as for standard *k*-means.

Like *k*-means++, this allows the initial centers to be spread as widely as possible and reduces the chances of significantly different peak profiles being incorporated into the same cluster. A secondary side-effect is that this method does not require the number of clusters, *k*, to be specified in advance, since in stage 5 we can stop center generation when the search space is adequately covered, using the condition *D*_*max*_ *< D*_*stop*_ with threshold parameter *D*_*stop*_, selected based on a preferred minimum cluster radius. It should be noted that since the mean is not a robust measure of central tendency, outlier observations should have ideally been removed prior to k-means clustering as these will affect both the clustering results and selection of the seed observation. In the case of k-medians, selection of an outlier as the seed is not inherently problematic, as the cluster center is repositioned appropriately in stage 7.

#### Cluster optimization

In the post-exploratory stages of analysis, several statistical metrics offer quantifiable and objective measures that can be used to optimize cluster analysis. The silhouette width [33] and Bayesian Information Criterion (BIC) [34] are common choices for assessing the results of clustering (see S1 File) for further details) and both are available in MetaboClust.

#### Annotation

Imported peak information, such as an adduct ion list, allows automated peak annotation to identify adducts or expected compounds in the metabolite data to produce a set of annotated peaks. The confidence level of these annotations can also be imported, for example, where identified only by accurate mass (as in the following case studies), the confidence level would be marked as "putative" [35], or confidence level 5 [36].

#### Pathway analysis

Pathway analysis allows the degree of overlap between pathways and clusters to be investigated. Peak annotation allows the number of compounds from any particular pathway that are present within a certain cluster to be determined. The resulting “cluster-pathway relationships” can be output to existing applications such as KEGG online or PathwayTools for visualization. Advanced batch correction methods, such as RUV [37] and COMBAT [38] are widely used in transcriptomic analyses. These techniques suffer several drawbacks when used in conjunction with metabolomic data [39,40] due to larger batch sizes and intra-batch drift. Additionally, RUV requires the factors of interest be stated up-front, making it unsuitable for use with unsupervised methods such as clustering. However, recent research has yielded variations more suited to unsupervised metabolomic analysis, including M-COMBAT [41] and RUV-2 [42]. Whilst the MetaboClust software focusses primarily on metabolomic, rather than transcriptomic, analysis, and cannot include the complete library of all available correction tools, an interface to the programming language, R, allows other tools for which a suitable package is present to be appropriated into the environment.

#### Implementation

Software implementation details are given in S2 File.

## Results and discussion

### Case study 1: Analysis of drought and disease in the model plant *Medicago truncatula*

The purpose of this study is to identify metabolites in *Medicago truncatula* responsive to the experimental conditions: drought (D), *Fusarium* infection (F) and the combination of both (B), in relation to the control group, (C). In particular, the aim is to highlight potential key pathways associated with the biotic and abiotic stress conditions. Biological replicates (plants) were extracted daily up to 13 days for each experimental condition. A pooled QC was introduced every 6^th^ sample in order to monitor instrumental noise. Experimental details are given in S3 File and the full dataset can be obtained from https://secure.fera.defra.gov.uk/abstress.

#### Data import

The MetaboClust import wizard was used to import the CSV files containing the *Medicago* peak intensities, as well as a list of compounds known to be present in *Medicago truncatula*. Here the peak intensity tables were obtained using Progenesis QI for peak picking. The compound list, which contains both hypothetical and known compounds, was downloaded from the MedicCyc database [43]. These data include information for 407 pathways, as well as mono-isotopic masses for the metabolites. A further CSV file containing mass and charge information for the eight possible adducts, shown in S1 Table, was also imported into the software. These 8 adducts represent the most frequently occurring adducts from the data published by Kind [44]. Annotations were assigned to peaks by performing accurate *m/z* matching against the imported metabolite and adduct databases. These annotations were therefore marked as "putative" [35] or confidence level 5 [36]. While MetaboClust allows more accurate compound "identifications" to be imported, due to the time and cost restraints, these are not available for our data. The list of file types accepted by MetaboClust for import is given S4 File.

#### Batch correction

**Fig 2:**
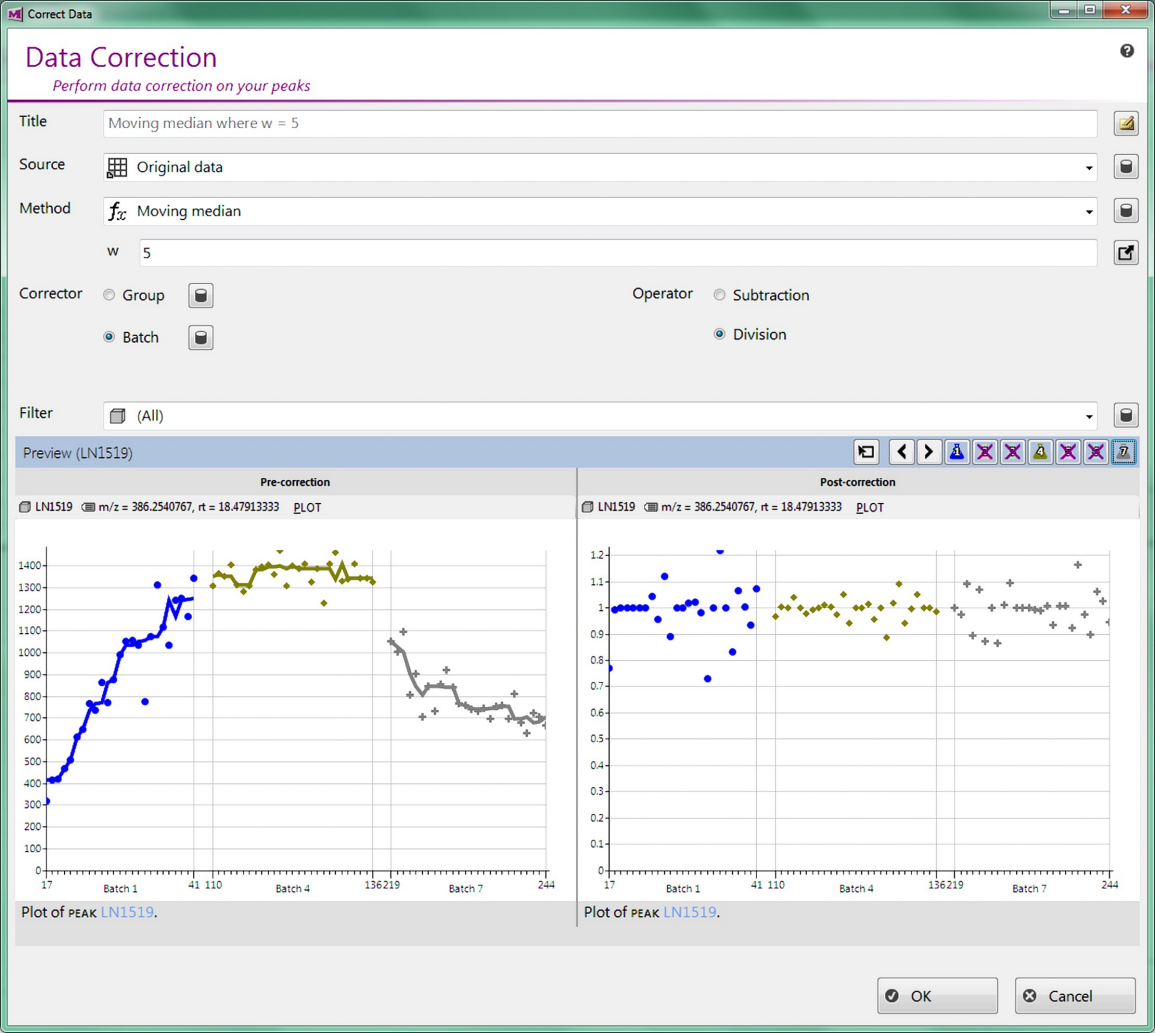
MetaboClust preview window showing background correction of a particular peak acquired in negative ionization mode. Compared to the QC corrected data (S3 Fig), variations in intensity (Y-axis), between batches and with acquisition order (X-axis) have been reduced.

Principle Components Analysis (PCA) (also available within the software) initially revealed notable differences between the LC-MS batches, with the between-batch variance overriding the variance between experimental groups. Signal correction using QC ion intensities for each batch [45] successfully resolved the batch differences in the positive ionization mode dataset. However, the MetaboClust visualization of the correction, shown in S3 Fig, makes it immediately apparent that the negative ionization mode data is not amenable to this correction. Subsequent PCA confirmed the unsuitability of the method and shows that the batch differences are exacerbated by QC-correction. For the negative data, we therefore used the “background correction” method, noted in the introduction, in lieu of using the QCs alone. The improved correction is shown in Fig 2. After batch correction, the data were scaled to unit variance and mean-centered, in order that all features be given equal weight in further analysis.

#### Control correction

It can initially be seen that a large number of peak profiles show a trend over time in the control-group. As it is the changes induced by the experimental conditions (*D*, *F*, and *B*) that are of primary interest, this trend was accounted for by applying control correction. The simplest way to produce a trend for the control group would be to average over the replicates at each time point. However, visualization in MetaboClust highlights a cause for concern as the method can transfer noise present in the control group to the other experimental groups. Here we found that a moving average was effective in keeping noise to a minimum while still accounting for the general trend of the control group. The median was selected as a more robust measure of central tendency over the mean, which in this case was overly sensitive to several outliers. A window width of 5 days was chosen to provide a smooth profile without significantly compromising resolution, this is depicted in S4 Fig.

#### Time profile generation

Noise removal and generation of the clustering input vectors was accomplished using a running average for smoothing, applied here as for control correction, i.e. a moving median with a window width of 5. A screen-shot showing time profile “trend” generation window is shown in S5 Fig.

#### Filtering

Initial, rapid clustering in MetaboClust reveals a number of clusters primarily dominated by noise. A filtering step was therefore included in our workflow, as discussed in the introduction. In-software classification of manual annotations (“clear-trend”, “no-clear-trend”, “undecided”) was used to optimize the *α* value, giving a 92% rate of match to the manual annotations at *α* = 0.082. An *α*-value slightly higher than usual (0.05) likely relates to the fact that early time-points typically show little change from the baseline, though we reiterate that the t-test is not being here used in the traditional sense to convey a statistical probability. Clustering results with and without filtering are discussed in section 3.1.7 below.

#### Optimization

Optimization of the number of clusters (*k*), shows that as *k* increases, the silhouette width shows a rapid decrease in performance, with the best clustering being performed for *k* = 2. The BIC performance statistic reveals similar results. This is indicative of a continuous spectrum of profiles, and unfortunately makes the decision on the number of clusters largely subjective. Whilst too many clusters make the identification of common patterns difficult, too few increase the complexity of individual clusters and thus fail to provide usable information. We therefore used *k = 25* as a reasonable compromise. On our data, both the results of the *k*-means algorithm and the deterministic variant of k-means++ (d-k-means++) produced very similar results, despite optimizing standard k-means over 1000 runs. At *k = 25*, the average deviation from the cluster center (*D*) for each metabolite was *D = 2*.*15* for d-k-means++ in comparison to *D = 2*.*11* for *k*-means. The closest 10% of metabolites have an average distance of *D*_*closest10*_ *= 1*.*11* with *k*-means and *D*_*closest10*_ *= 1*.*07* for d-k-means++. Since the differences between two methods are small we therefore favored the d-k-means++ algorithm for the remainder of this analysis, over the much slower *k*-means.

#### Clustering

**Fig 3:**
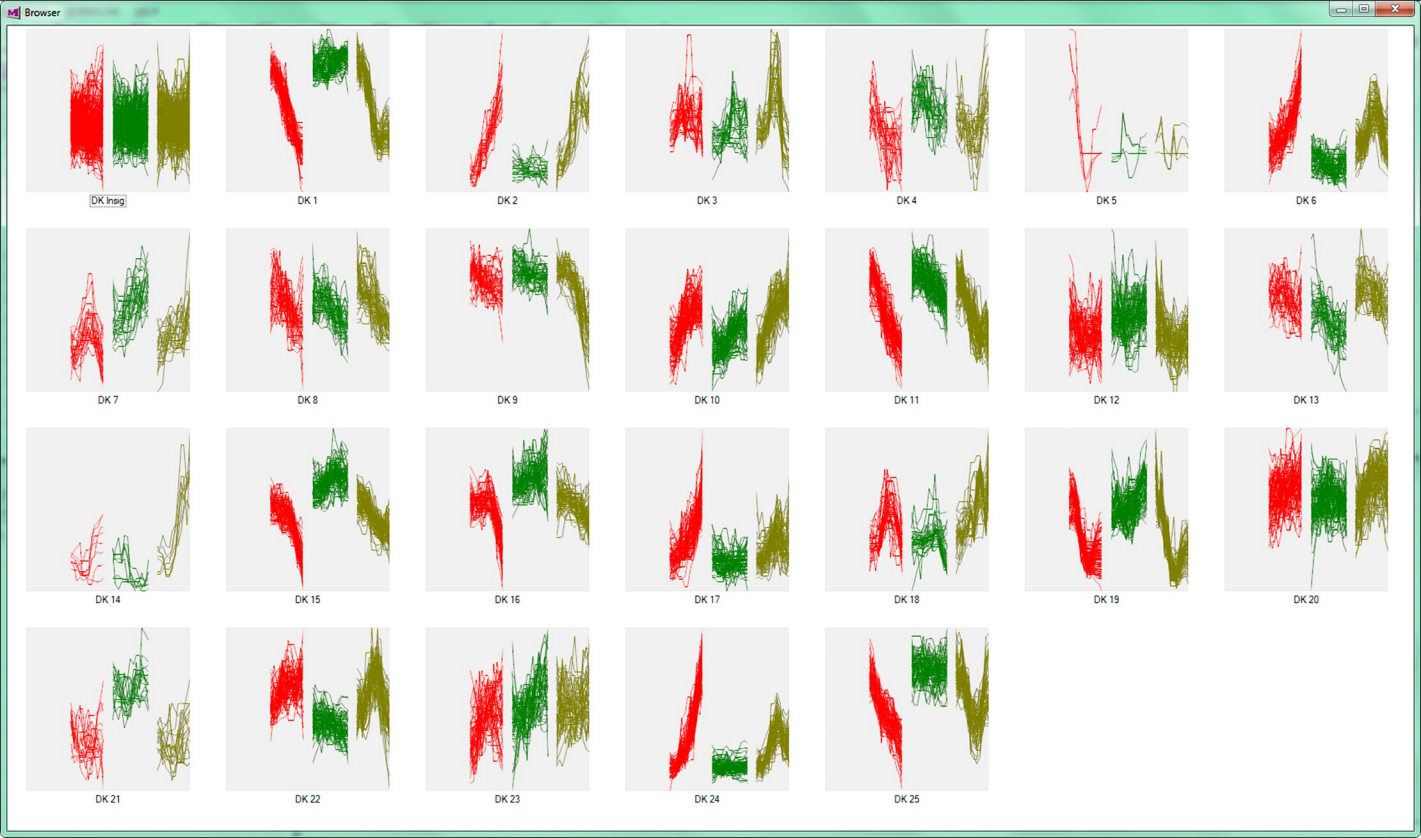
Screen-shot showing thumbnail views of the 25 clusters obtained for the Medicago dataset after filtering. Input vectors are colored by experimental group). The-X axis corresponds to the input vectors, organised as drought group, days 2–13 (red); Fusarium group, days 1–13 (green); Dual-stress group, days 2–13 (yellow). The Y-axis corresponds to the auto scaled peak intensity.

**Fig 4:**
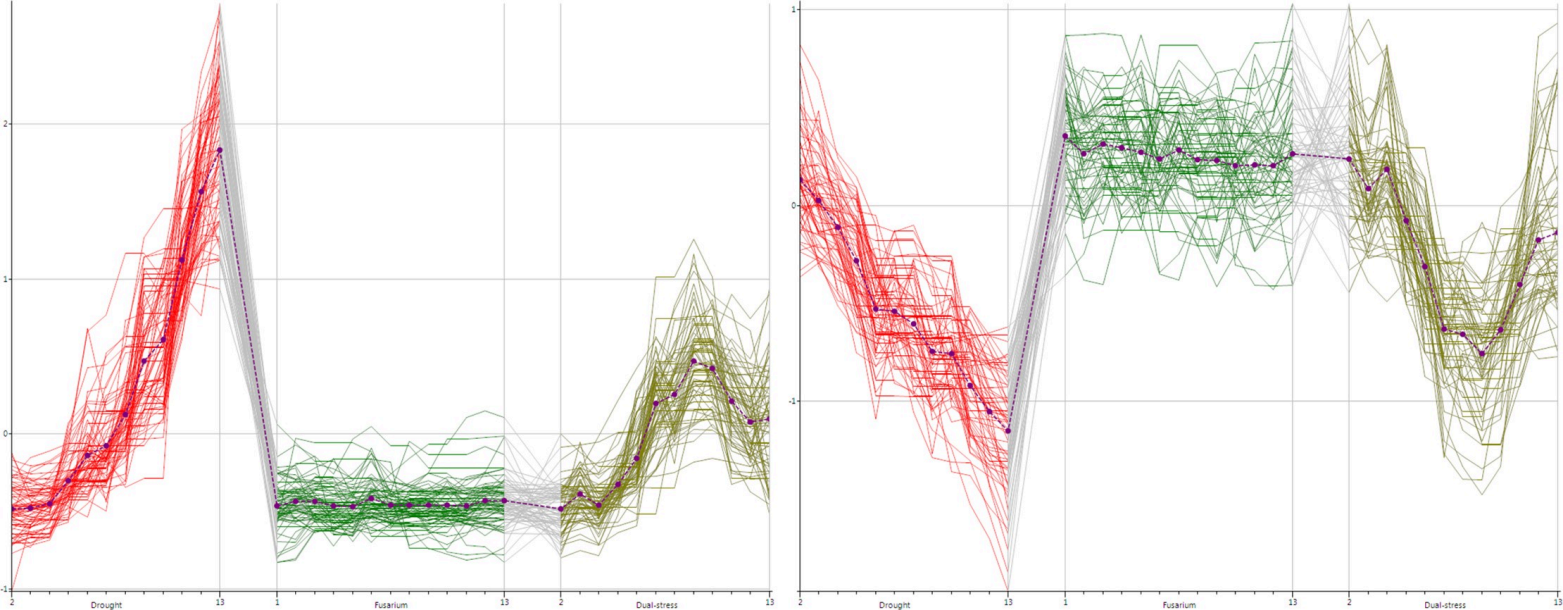
Close-up of thumbnail views of clusters 24 and 25. Taken from the 25 clusters obtained for the Medicago dataset in MetaboClust’s cluster explorer (Fig 3). Axes and colors are the same as those presented in Fig 3.

Visualization of the clustering results is shown in Fig 3. Whilst differences between the control and *Fusarium* groups were not apparent during the earlier exploration of individual time-points, cluster analysis was able to reveal time profiles that differed between the two groups, such as can be seen in clusters 7 and 8. Substantial differences between the profiles of drought and dual stressed plants are also present, such as clusters 24 and 25, which are shown enlarged in Fig 4. Fig 3 provides a summary view of the time profiles of our dataset, allowing the operator to select clusters of interest and also acts as a quick overview of clustering performance. S6 Fig shows the results of clustering without the filtering stage.

#### Pathway analysis

Cluster 18 shows a group of stress responsive compounds that increase in intensity over time for the dual-stress group, whilst dropping in intensity for the drought and Fusarium groups. This cluster contains 36 input vectors representing 36 peaks, of which 15 have been assigned tentative compound identifications. The compounds touch upon several pathways, with the highest degree of overlap being the “tRNA charging” (protein biosynthesis) pathway, comprising the set of the 20 standard amino acids. The accumulation of amino acids in drought stressed plants is well known from the literature (24–26) The decreasing concentrations of potential amino acids seen in the dually stressed group likely reflect a failure of the stress coping mechanisms and warrant further investigation.

**Fig 5:**
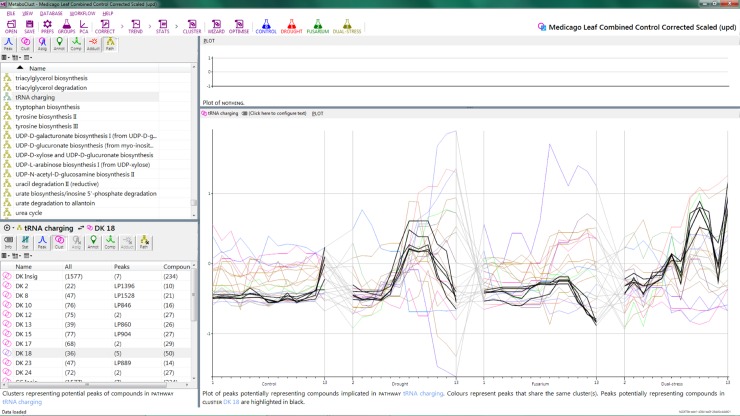
Screen-shot the time profiles for cluster and pathway overlaid. Here cluster 18 and the tRNA charging pathway have been chosen so that all profiles in cluster 18 are shown with those in bold having tentative annotations that could be associated with the tRNA charging pathway.

Another notable cluster, Cluster 19 shows a group of features with very similar time profiles for the drought and dual-stress groups. This cluster comprises 87 peaks and 9 shows overlap with the TCA Cycle (29 compounds), depicted in Fig 5. The TCA cycle is known to be down-regulated during drought-stress, likely as a consequence of stress rather than as an adaptive benefit [46].

Pathway cluster relationships can be exported from MetaboClust in the form of compound lists identified for each pathway. These can then be imported into the MetaCyc online pathway browser to highlight the compounds in the pathway perturbed by experimental conditions. An example MetaCyc pathway view is shown in S7 Fig. with compounds corresponding to annotated peaks in cluster 2 highlighted.

### Case study 2: Comparison of phenotypes of *Alopecurus myosuroides*

*Alopecurus myosuroides* is an important grass weed, known in the United Kingdom as “Black Grass”, it affects arable yield via competition with the crop species. A major control mechanism for these grasses is the use of herbicides and, consequentiality, a number of herbicide resistant varieties of Black Grass have been reported in recent years [47,48]. Identification of the chemical families indicative of resistance type would facilitate diagnostics and avoid unnecessary use of ineffective herbicides. In this study, three *A*. *myosuroides* phenotypes were grown and analyzed at days 0, 4, 8 and 13. The phenotypes include susceptible plants (S), vulnerable to herbicide use; target site resistant plants (T), tolerant of specific herbicide use; and multiple herbicide resistant plants (M), resistant against a diverse range of herbicide families. Full experimental details are given in S3 File.

#### Data import

As no database specific to *Alopecurus* could be found, databases for several different plant species were downloaded from the PMN plant metabolic pathway database collection in order to cover as many metabolites as possible. These are shown in S2 Table. All are available in the PathwayTools database format, which can be imported into MetaboClust. We use this approach to provide immediate links with pathway ontologies. Whilst we acknowledge this is speculative in terms of type 1 errors [49], this method allows for a reasonable glimpse of the metabolic makeup of our dataset as a whole. A preferable, though extensively time-consuming approach would be to use dedicated databases, such as ReSpect (http://spectra.psc.riken.jp) or GNPS (https://gnps.ucsd.edu) and then associate metabolites using reconstruction networks of the known pathways of the closest species. As in the *Medicago study*, the *Alopecurus* data and the adduct list were imported as CSV files.

#### Batch correction and control correction

Peak intensities were UV scaled and mean centered in software. Neither batch nor control correction was possible as all data were acquired in one batch per ionization mode and no control group was available.

#### Time-profile generation

Using the MetaboClust visualization to determine the effectiveness of different smoothing algorithms, we found that taking the median of the replicates for each time point was sufficient to generate time profiles. In contrast to the noisier *Medicago* dataset, with more time-points, a more complex smoothing function was not required. However, since the response of individual species in this case is of interest, a separate clustering vector was generated for each experimental group, as opposed to the single vector formed by concatenation of the experimental groups, as used in the *Medicago* study.

#### Optimization

Whilst BIC again favored a minimal number of clusters (*k = 2*) it did not show a gradual decrease in clustering performance with *k*, instead presenting a second peak in performance at *k* = 10, which we therefore used as *k* in further analysis. This value of *k* was again consistent between d-k-means++ and optimized k-means, for these data. S8 Fig shows the cluster optimization using BIC.

#### Clustering

**Fig 6:**
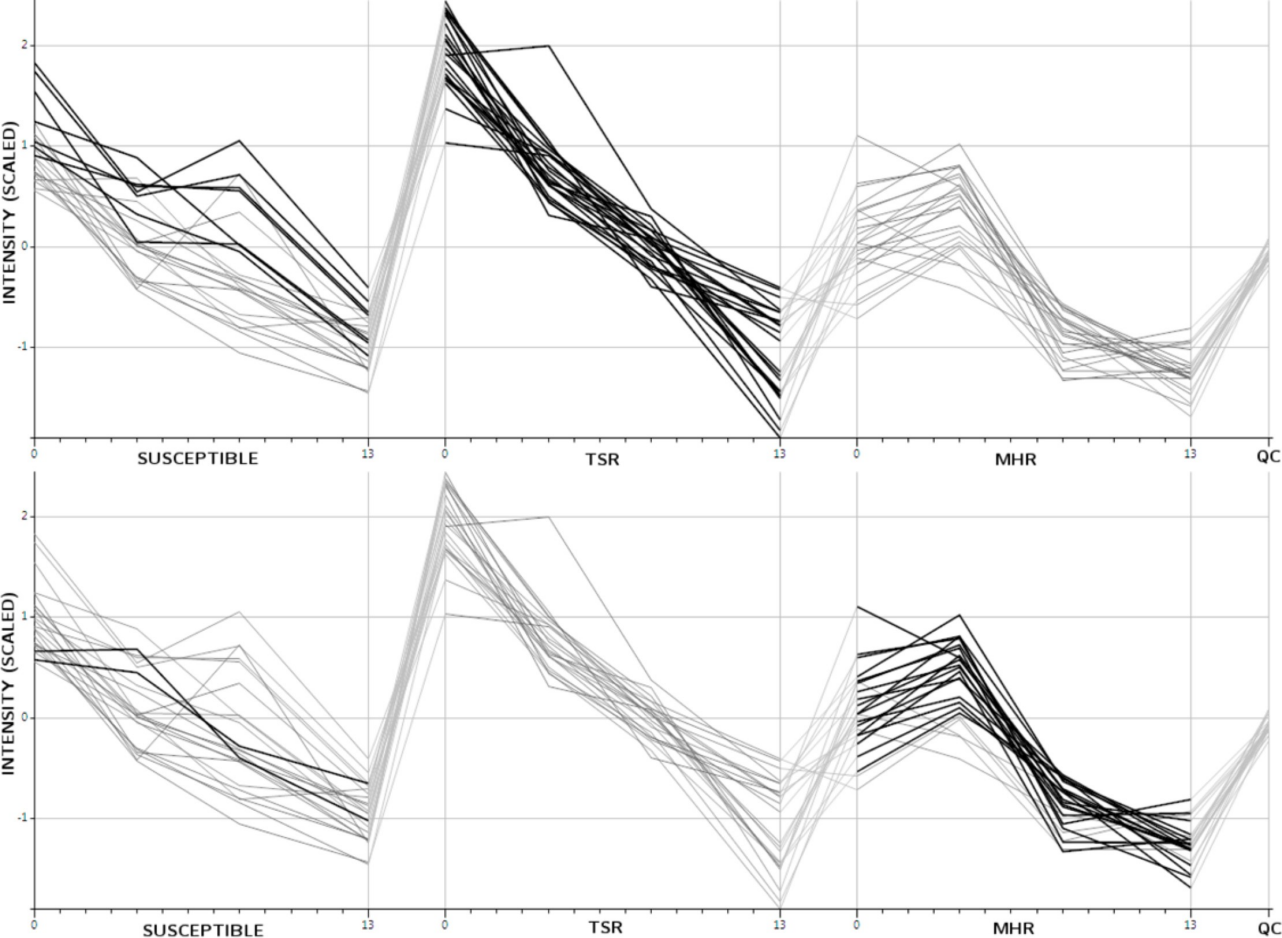
Time profiles for peaks potentially representing compounds of the brassinosteroid biosynthesis pathway. Profiles for each group were considered separately. The upper panel shows those time-profiles that group together in cluster 2 in bold (mainly from the T group) and the lower panel shows those that group together in cluster 3 in bold (mainly the M group with a couple from the susceptible group).

A number of interesting patterns can be observed using group-wise clustering. Cluster 2 includes profiles showing a decrease with time. The pathway breakdown, shown in Table 1, notes *Brassinosteroid* biosynthesis as being indicative of this cluster. Brassinosteroids are a class of hormonal regulators, implicated in the plant stress response [50]. Fig 6 shows the time profiles in our dataset that potentially represent compounds in the brassinosteroid biosynthesis pathway, with those in cluster 2 shown in bold. It can be seen that cluster 2 primarily overlaps with the T group, with the S group being represented by a similar downward trend. It is interesting to note that the multiple herbicide resistance group members (M) do not show the same continual decrease in these compounds over their lifetime and the diverging nature of these groups provides an avenue for further research.

**Table 1:** Results of pathway analysis on the time profiles in Case Study 2. The number of peaks potentially be associated with each of the top five pathways in cluster 2 is shown. Compounds are listed as "potential" as they have been matched against putative annotations only and further experimental analysis is required to confirm their presence. As multiple peaks could represent the same compound, the number of compounds in the pathway with annotations in cluster 2 is also given.

Pathway	Number of peaks	Number of potential compounds
brassinosteroid biosynthesis I	23	7 (6-deoxotyphasterol, 6-deoxoteasterone, 6-oxocampestanol, castasterone, 3-dehydroteasterone, teasterone, typhasterol)
simple coumarins biosynthesis	22	6 (ferulate, herniarin, 4-coumarate, umbelliferone, shikimate, S-adenosyl-L-methionine)
plant sterol biosynthesis II	20	8 (4α-carboxy,4β,14α-dimethyl-9β,19-cyclo-5α-cholest-24-en-3β-ol, 4α-carboxy-5α-cholesta-7,24-dien-3β-ol, 4α-formyl-5α-cholesta-7,24-dien-3β-ol, 4α-hydroxymethyl-5α-cholesta-7,24-dien-3β-ol, 5α-cholesta-7,24-dien-3β-ol, desmosterol, 7-dehydrodesmosterol, 5α-cholesta-7,24-dien-3-one)
phenylpropanoid biosynthesis	20	8 (5-hydroxy-coniferaldehyde, coumaraldhyde, 4-coumarate, L-quinate, shikimate, sinapaldehyde, S-adenosyl-L-methionine, trans-5-O-(4-coumaroyl)-D-quinate)
suberin biosynthesis	20	7 (18-oxo-oleate, ferulate, trans-cinnamate, 4-coumarate, S-adenosyl-L-methionine, 22-hydroxydocosanoate, docosanedioate)

## Conclusions

Unlike script or web-based tools, MetaboClust permits simple and immediate access to statistical information and interactive visualizations, allowing the user to rapidly test different processing methods. Various options for data correction and statistical analysis are available to the user and can be used interactively. In particular, the user can try different approaches and accept or reject the results before moving on to the next stage. This is also of potential use to experimental biologists as MetaboClust can be used to rapidly review acquired data. We believe that there is no best scenario for analysis from batch correction through to pathway analysis and that choices, even whether or not to scale the data, need to be informed by exploratory analyses. Our aim with MetaboClust is to not only to provide a novel clustering pipeline, but also to facilitate this interactive exploratory analysis and allow the user to return to any stage in the data analysis without having to begin again from the raw data.

Existing tools for metabolomic analysis largely terminate by assigning scores to the peaks or observations submitted for analyis. Whilst some do offer cluster analysis, the resulting clusters are typically the end-point rather than the objects to be analyzed. As far as we are aware, MetaboClust is the only software tool that considers metabolomic time-course clustering in a cohesive workflow with emphasis on the clusters themselves as a point of analysis. MetaboClust allows a coherent pipeline to clustering: while both script and web-based software tools require their workflows to be designed by the user in advance, either in the form of a script or by submitting a "job" to the server, such a process inhibits an exploratory analysis. We have sought to address this issue by retaining the workflow in memory, allowing the user to see how changes in one step of the analysis will affect others further down the line and permitting changes to the parameters of the analysis as issues become apparent. Rich, interactive visualization presented during the analysis itself, rather than solely as an output assists in this task. MetaboClust is the only software to include the QC-independent “background” batch correction of metabolomic data, and presents a visual representation of this, as well as other batch correction methods. Additionally, should the user desire, use of a custom, deterministic variant of k-means allows changes in parameters to be more easily and quickly explored.

There are some disadvantages intrinsic to this methodology. The tight focus on clustering does mean that complementary software is required for other stages of analysis, such as peak-picking. The focus on standard batch correction and background correction methods means that more esoteric tools are not available within Metaboclust, though the interface to R alleviates this issue by allowing access to R’s large collection of software libraries. The implementation of a GUI incurs common drawbacks: notably GUI-based software tools are known to be slower than script based ones if a pre-determined workflow is to be followed and the user is familiar with the software. However, script-based tools do typically have a higher learning curve, making them less accessible to users at the point of data collection. GUI based tools such as MetaboClust, therefore can serve either as the primary mechanism for analysis or as a forerunner to a well-defined script, once the algorithms and parameters can be settled upon by the user.

Using two datasets as case studies we have shown that MetaboClust can provide new insight in metabolomics. Using workflows constructed both visually and through within-software parameter optimization, differences were identified between groups that were not apparent without time-series analysis. Alongside clustering, pathway information from external databases allowed notable response profiles to be linked to pathways of interest. While the annotations used in our case studies were estimated using accurate mass only and therefore represent confidence level 5 [36] or "putative" identifications, the combination of multiple tentative assignments allowed several pathway perturbations to be proposed that are supported by the current literature. These identifications act as a viable focal point from which more definitive metabolite assignments can be made and pathways confirmed.

MetaboClust is released under the GPLv3 (https://www.gnu.org/licenses/gpl-3.0), an open-source license, allowing free use of the code and component libraries.

## Supporting information

S1 File Document providing details on cluster optimization using the silhouette width and BIC. (DOCX)

S2 File Document providing details on the design of the software. (DOCX)

S3 File Document providing a description of the data and experimental methods. (DOCX)

S4 File Document describing the input data. (DOCX)

S1 Table List of adducts used for *m/z* based peak annotation in the *Medicago* case study. (DOCX)

S2 Table Databases imported into MetaboClust for the *Alopecurus* case study. (DOCX)

S1 Fig Visual abstraction of the process. The workflow allows a set of clusters to be generated, representing metabolite concentrations affected by the experimental conditions. Perturbed pathways are suggested by the software, allowing the user to export their data into pathway analysis tools such as MetaCyc (online). Since the workflow is unlikely to be known upfront, the user is actively involved in all stages of analysis.
(TIF)

S2 FigSimple example to explain batch correction.(a) For peak *p*, the original intensities *X*_*p*,*b*,*i*_ for QC samples are indicated by black points and experimental samples are shown in colour. The increasing trend seen in the experimental samples is also evident in the QCs and can be modelled by the regression line obtained from the QCs. The correction factor *C*_*p*,*b*,*i*_ in this case is gven by the values predicted by the regression for both QCs and experimental samples. (b) shows the intensities after subtraction of the correction factors with negative values for samples below the regression line. In (c) the horizontal line represents *R*_*p*_, here taken to be the median value of the original QC intensities, which is then added to obtain the corrected intensities $X_{\;p,b,i}^{new}=X_{p,b,i}-C_{p,b,i}+R_{p}$ shown.(TIF)

S3 Fig MetaboClust preview window showing mean-of-the-QCs batch correction of a particular peak acquired in negative ionization mode. Variations in intensity (Y-axis), both between batches and along the acquisition order (X-axis), can be seen pre-correction (left) as well as post-correction (right) showing that correction is not achieved using this method.
(TIF)

S4 Fig Preview window showing control correction of a particular peak. Variations in intensity (Y-axis) due to growth (time, X-axis) can be seen pre-correction (left). Subtraction of the control profile (right) allows the analysis to concentrate on deviations from this profile. Using a smooth trend for the profile avoids the tranfer of noise from the control group into the other experimental groups.
(TIF)

S5 Fig Screen-shot showing time profile “trend” generation window. The experimental observations are displayed on the graph, for each experimental group in turn. Blue = control, red = drought stress, green = *Fusarium* stress, yellow = dual stress. The X axis corresponds to day, and the Y axis to signal intensity. The bold line shows the trend that will be generated for the current settings, in this case a moving median with a window width of 5.
(TIF)

S6 Fig Screen-shot showing thumbnail views of the 25 clusters obtained for the Medicago dataset without filtering. The effects of not filtering the data are apparent in a number of clusters with noisy profiles. Other clusters are also affected by the presence of erratic time profiles. The-X axis corresponds to the input vectors, laid out as drought group, days 2–13 (red); Fusarium group, days 1–13 (green); Dual-stress group, days 2–13 (yellow). The Y-axis corresponds to the auto scaled peak intensity.
(TIF)

S7 Fig MetaCyc pathway view. Pathway cluster relationships can be exported from MetaboClust and imported into the MetaCyc online pathway browser. This allows the compounds perturbed by experimental conditions to be highlighted in the pathway. Here the compounds corresponding to annotated peaks in cluster 2 are highlighted by the solid red circles.
(TIF)

S8 Fig Clustering performance measured using BIC. BIC is plotted on the Y axis against the number of clusters, *k*, on the X axis. Excluding *k* = 2 the optimal number of clusters is shown to be *k* = 10.
(TIF)
