# Supplementary material for: MetaboClust: Using interactive time-series cluster analysis to relate metabolomic data with perturbed pathways
Source: PLoS One. 2018 Oct 29;13(10):e0205968. doi: 10.1371/journal.pone.0205968 (PMC6205582; doi:10.1371/journal.pone.0205968)
Supplement: S1 File — (DOCX) [file pone.0205968.s001.docx]

# Optimisation

We have used two methods of optimizing the number of clusters, the silhouette width and the Bayesian Information Criterion (BIC). The silhouette width shows how well a point fits into its assigned cluster in relation to how well it might fit into a different cluster, it is defined as:

$$s\left( i \right)=\frac{b\left( i \right)-a(i)}{max\{a\left( i \right),b\left( i \right)]}$$

Here,

$$a\left( i \right)=d(i,g\left( i \right))$$

and

$$b\left( i \right)={min}_{i\neq j}d(i,g\left( j \right))$$

where *d(i,g(j))* is the average distance (dissimilarity) from object *x_i_* to all other points in a cluster:

$$d\left( i,g\left( j \right) \right)={average}_{x_{j}\epsilon g(j)}|x_{i}-x_{j}|$$

Thus, *a(i)* is a measure of how well object *x_i_* fits into its assigned cluster, and *b(i)* is a measure of how well object *x_i_* fits into the next-best possible cluster. Now -1 ≤ s(i) ≤ 1 and values closer to 1 indicate better assignments whereas values close to -1 indicate an object that would be better suited to a different cluster. Values close to zero indicate objects on the border of two clusters. The values of *s(i)* can be averaged over a group of objects, such as those within a cluster in order to determine how tightly the data is clustered and the average over the entire dataset provides a measure of clustering performance.

The BIC provides an alternative performance metric that, unlike the silhouette width, applies a penalty for results with more clusters:

$$BIC=-2\ln\left( L \right)+k\ln(n)$$

Here *k* is the number of free parameters, in this case the number of clusters, *n* is the number of observations, and *L* is the value of the maximum likelihood function.
