# Supplementary material for: MetaboClust: Using interactive time-series cluster analysis to relate metabolomic data with perturbed pathways
Source: PLoS One. 2018 Oct 29;13(10):e0205968. doi: 10.1371/journal.pone.0205968 (PMC6205582; doi:10.1371/journal.pone.0205968)
Supplement: S2 File — (DOCX) [file pone.0205968.s002.docx]

# The Software

MetaboClust software supports the user in creating and exploring time series profiles and to identify similar profiles and potential metabolic pathways they relate to.

The software was developed using Microsoft C# and the .NET framework, which provides a library of reusable classes (the Framework Class Library). In particular, we make use of the Windows Forms library, which provides an out-of-the-box means to interactively and iteratively develop a user-friendly interface. Linking between programming languages is facilitated by .NET and we use the R.NET library to provide an in-process interoperability bridge to the R script interpreter from .NET [1]. The use of R allows the incorporation of further statistical analysis, as well as allowing users to incorporate their own methods and providing easy access to newly developed algorithms. A number of mathematical functions, such as the distance metrics, make use of the Math.NET Numerics package [2].

Data is imported into a session via a guided importation wizard. All data files are expected to be in CSV format due to wide support format in existing conversion utilities. After a session has been created it is saved by the software into a native binary format for speed of access.

# References

1. Abe K, Perraud J-M. R.NET. NuGet (Microsoft, .NET Foundation); 2015.

2. Rüegg C. Math.NET Numerics. NuGet (Microsoft, .NET Foundation); 2015.
