# Supplementary material for: MetaboClust: Using interactive time-series cluster analysis to relate metabolomic data with perturbed pathways
Source: PLoS One. 2018 Oct 29;13(10):e0205968. doi: 10.1371/journal.pone.0205968 (PMC6205582; doi:10.1371/journal.pone.0205968)
Supplement: S3 File — (DOCX) [file pone.0205968.s003.docx]

# Experimental data

Two datasets are used as case studies to demonstrate use of the MetaboClust software.

## Medicago data set

The *Medicago* dataset constitutes a time-course study on the effects of biotic and abiotic stress in *Medicago truncatula*, a model legume. Full details of sample preparation and data acquisition are given in [1]. Three* biological replicates (plants) were extracted daily for each of the following four experimental conditions:

- C – Control plants (no stress), harvested days 0-12.
- D – Plants subject to abiotic stress (drought), harvested days 1-12.
- F – Plants subject to biotic stress (infected with the pathogen *Fusarium oxysporum*), harvested days 0-12
- B – Plants subject to dual stress (subject to both drought and infection with *Fusarium oxysporum*), harvested days 1-12.

(* although three replicates for each condition were available for most days, four droughted replicates were available for day 2 and only two control replicates were available for days 5 and 12.)

For each replicate, leaf samples were taken from the extracted plants and analysed using Liquid Chromatography – High Resolution Mass Spectrometry (LC-HRMS) in both positive and negative ionization mode. Alignment and peak picking was performed using Progenesis QI (Waters Corporation, Milford, MA, USA), providing a matrix of integrated peak intensities for each observation. The features represent integrated intensity (peak areas) for plot regions described by their mass to charge ratio (*m/z*) (between 80 and 1000) and retention time (between 1 and 30 minutes). The data from both positive and negative ionization modes was consolidated to produce the final dataset, comprising a total of 2920 features (1681 positive and 1239 negative) for 184 observations (149 experimental and 35 QC). However, some of these features may constitute noise or artefacts of the peak-picking algorithm and several peaks may also represent the same compound.

## Alopecurus data set

The *Alopecurus* dataset involves a short time series with just 4 discrete time points for a study of herbicide resistance in the grass weed *Alopecurus myosuroides* for which a number of herbicide resistant varieties have been reported in recent years [2,3]*.*

Plants were analysed at days 0, 4, 8 and 13, for each of the following three phenotypes of the grass:

- S – Susceptible plants, vulnerable to herbicide use.
- TSR (T) – Target site resistant plants, tolerant of specific herbicide use.
- MHR (M) – Multiple herbicide resistant plants, resistant against multiple herbicide families.

Alignment and peak picking was performed using Progenesis QI as with the *Medicago* data.

### Plant materials and growth conditions

MHR (Essex, UK.), TSR (Nottingham, UK.) and Susceptible (Rothamsted, UK.) seed lines of *Alopecurus myosuroides* were planted into 12 cm diameter terracotta pots in a peat based compost (Petersfield, Cambridge, UK.) using 20 seeds per pot. Plants (n=3 per seed line) were grown under controlled glasshouse conditions at Fera Science Ltd (Fera, North Yorkshire, UK). There were 24 plants in total which contained 3 replicates for each line over 4 time points - Day 0 (pre-treated samples), Day 4 (post spray), Day 8 and Day 12. After 3 weeks of growth, plants were sprayed at field rates with Topik (an acetyl co A carboxylase inhibitor) and harvested directly into liquid nitrogen cutting from just above the soil line.

### Metabolite extraction

Each frozen plant was lyophilized overnight and ground into a fine powder using an A 11 basic analytical mill (IKA, Staufen, Germany). In a labeled 2 mL eppendorf tube, 1ml of extraction solvent (1:1 (v/v) methanol: water) was added to 5 mg of ground sample. Metabolites were extracted into the solvent by shaking for 30 minutes. The solid material was then removed by centrifugation at 14,000 rpm for 10 minutes at ambient temperature. To prepare samples for profiling analysis by LC-HRMS the supernatant was diluted 9:1 with 1:1 (v/v) methanol: water. An analytical quality control (QC) sample was created by pooling 1 ml from each final sample extract.

### LC-HRMS profiling conditions

LC analysis was performed on an Accela 1250 High Speed LC system from Thermo Fisher Scientific (Waltham, Massachusetts, USA). The analytical column used was an ACE Excel AQ (Advanced Chromatography Technologies, UK) 150 mm x 3 mm, 100 Å. Mobile phase A (MPA) was 0.1% formic acid in HPLC water, mobile phase B (MPB) was 0.1% formic acid in acetonitrile. A linear gradient elution was applied over 10 minutes from 100% MPA to 100% MPB. The gradient was then held for 2 minutes at 100% MPB before re-equilibration with 100% MPA for a further 2 minutes. The LC flow rate was 0.4 mL min^-1^ and the column temperature was 30°C. Sample injection volume was 5 μL. The MS used was an Orbitrap Velos Pro hybrid ion trap high resolution mass spectrometer (Thermo Fisher Scientific, Waltham, Massachusetts, USA) with a mass resolution setting of 60,000 at *m/z* 200. Maximum injection time was 50 ms. Ionization was by heated electrospray (HESI) with extracts analyzed in both positive and negative mode. The source heater temperature was set to 450 °C with sheath gas set to 51 and aux gas at 16 (au, arbitrary units). The capillary temperature was 370°C.

Sample analysis order was randomized using www.random.org. Internal standard was not added to the samples. Signal normalization to correct for signal change due to the length of time of the run was undertaken using the QC (a pooled extract containing an aliquot which was analyzed every 6 samples). This assumes that every signal (e.g. feature or m/z–RT combination) is present in the pooled QC.

# References

1. Rusilowicz M, Dickinson M, Charlton A, O’Keefe S, Wilson J. A batch correction method for liquid chromatography–mass spectrometry data that does not depend on quality control samples. Metabolomics. 2016;12: 1–11.

2. Cummins I, Edwards R. The Biochemistry of Herbicide Resistance in Weeds. Outlook Pest Man. Research Information; 2010;21: 73–77.

3. Chauvel B, Guillemin JP, Colbach N, Gasquez J. Evaluation of cropping systems for management of herbicide-resistant populations of blackgrass (Alopecurus myosuroides Huds.). Crop Protection. 2001;20: 127–137.
