# Supplementary material for: MetaboClust: Using interactive time-series cluster analysis to relate metabolomic data with perturbed pathways
Source: PLoS One. 2018 Oct 29;13(10):e0205968. doi: 10.1371/journal.pone.0205968 (PMC6205582; doi:10.1371/journal.pone.0205968)
Supplement: S4 File — (DOCX) [file pone.0205968.s004.docx]

# Accepted import files

- **Intensity matrix** – a data matrix with rows corresponding to observations and columns corresponding to integrated peak intensities.
- **Observation data** – details on experimental observations are required for statistical analysis, including the *experimental group*, *time-point* and *replicate number* of each observation.
- **Peak data** – *m/z* values can be imported to allow peak annotations to be made using the metabolite database. Peak annotations (obtained for example via Progenesis QI) can also be loaded to replace or augment the automated annotations.
- **Metabolite database** – information on metabolites (name and *m/z*) is required for automated annotation and details of relevant metabolic pathways are required for pathway analysis. The software is able to import databases in the BioPAX pathway exchange format [1], in addition to providing the data as a spreadsheet (CSV).
- **Adduct database** –a list of potential ion adducts is required if automated identification of LC-HRMS data is to be implemented.
- **Extensions** – At all stages of analysis external R scripts can be implemented where more esoteric solutions are desired. Custom filters are also supported and can be used to exclude specific observations or isolate interfering features, such as outliers or noise.

**References**

1. Demir E, Cary MP, Paley S, Fukuda K, Lemer C, Vastrik I, et al. The BioPAX community standard for pathway data sharing. Nature Biotechnology. 2010 Sep 9;28(9):935–42.
