# Supplementary material for: MetaboClust: Using interactive time-series cluster analysis to relate metabolomic data with perturbed pathways
Source: PLoS One. 2018 Oct 29;13(10):e0205968. doi: 10.1371/journal.pone.0205968 (PMC6205582; doi:10.1371/journal.pone.0205968)
Supplement: S1 Table — (DOCX) [file pone.0205968.s005.docx]

| **Adduct** | **Charge** | **Mass Difference (M)** |
| --- | --- | --- |
| -H | −1 | M−1*.*00728 |
| -2H | −2 | M/2−1*.*00728 |
| -H_2_O-H | −1 | M−19*.*0184 |
| +H | +1 | M+1*.*007276 |
| +Na | +1 | M+22*.*98922 |
| +K | +1 | M+38*.*96316 |
| +NH_4_ | +1 | M+18*.*03382 |
| +2H | +2 | M/2+1*.*007276 |

S1 Table. List of adducts used for *m/z* based peak annotation in the *Medicago* case study. These represent a subset of those found in [1].

# References

1. Kind T. Mass Spectrometry Adduct Calculator. University of California; 2010.
