# Supplementary material for: MetaboClust: Using interactive time-series cluster analysis to relate metabolomic data with perturbed pathways
Source: PLoS One. 2018 Oct 29;13(10):e0205968. doi: 10.1371/journal.pone.0205968 (PMC6205582; doi:10.1371/journal.pone.0205968)
Supplement: S2 Table — (DOCX) [file pone.0205968.s006.docx]

| AraCyc 13.0 | BarleyCyc 3.0 | BrachypodiumCyc 3.0 |
| --- | --- | --- |
| CassavaCyc 5.0 | ChineseCabbageCyc 3.0 | ChlamyCyc 5.0 |
| CornCyc 6.0 | GrapeCyc 5.0 | MossCyc 4.0 |
| OryzaCyc 3.0 | PapayaCyc 4.0 | PoplarCyc 8.0 |
| PotatoCyc 2.0 | SelaginellaCyc 4.0 | SetariaCyc 3.0 |
| SorghumBicolorCyc 3.0 | SoyCyc 6.0 | SpirodelaCyc 1.0 |
| SwitchgrassCyc 3.0 | TomatoCyc 1.0 | WheatACyc 1.0 |
| WheatDCyc 1.0 | MedicCyc* |  |

S2 Table. Databases imported into MetaboClust for the *Alopecurus* case study. *With the exception of the MedicCyc *Medicago* database [1], these were downloaded from the PMN database collection [2].

# References

1. Caspi R, Foerster H, Fulcher CA, Kaipa P, Krummenacker M, Latendresse M, et al. The MetaCyc Database of metabolic pathways and enzymes and the BioCyc collection of Pathway/Genome Databases. Nucleic acids research. 2008;36: D623–D631.

2. Zhang P, Dreher K, Karthikeyan A, Chi A, Pujar A, Caspi R, et al. Creation of a genome-wide metabolic pathway database for Populus trichocarpa using a new approach for reconstruction and curation of metabolic pathways for plants. Plant Physiology. 2010;153: 1479–1491.
