# Supplementary figures and images for: MetaboClust: Using interactive time-series cluster analysis to relate metabolomic data with perturbed pathways
Source: PLoS One. 2018 Oct 29;13(10):e0205968. doi: 10.1371/journal.pone.0205968 (PMC6205582; doi:10.1371/journal.pone.0205968)

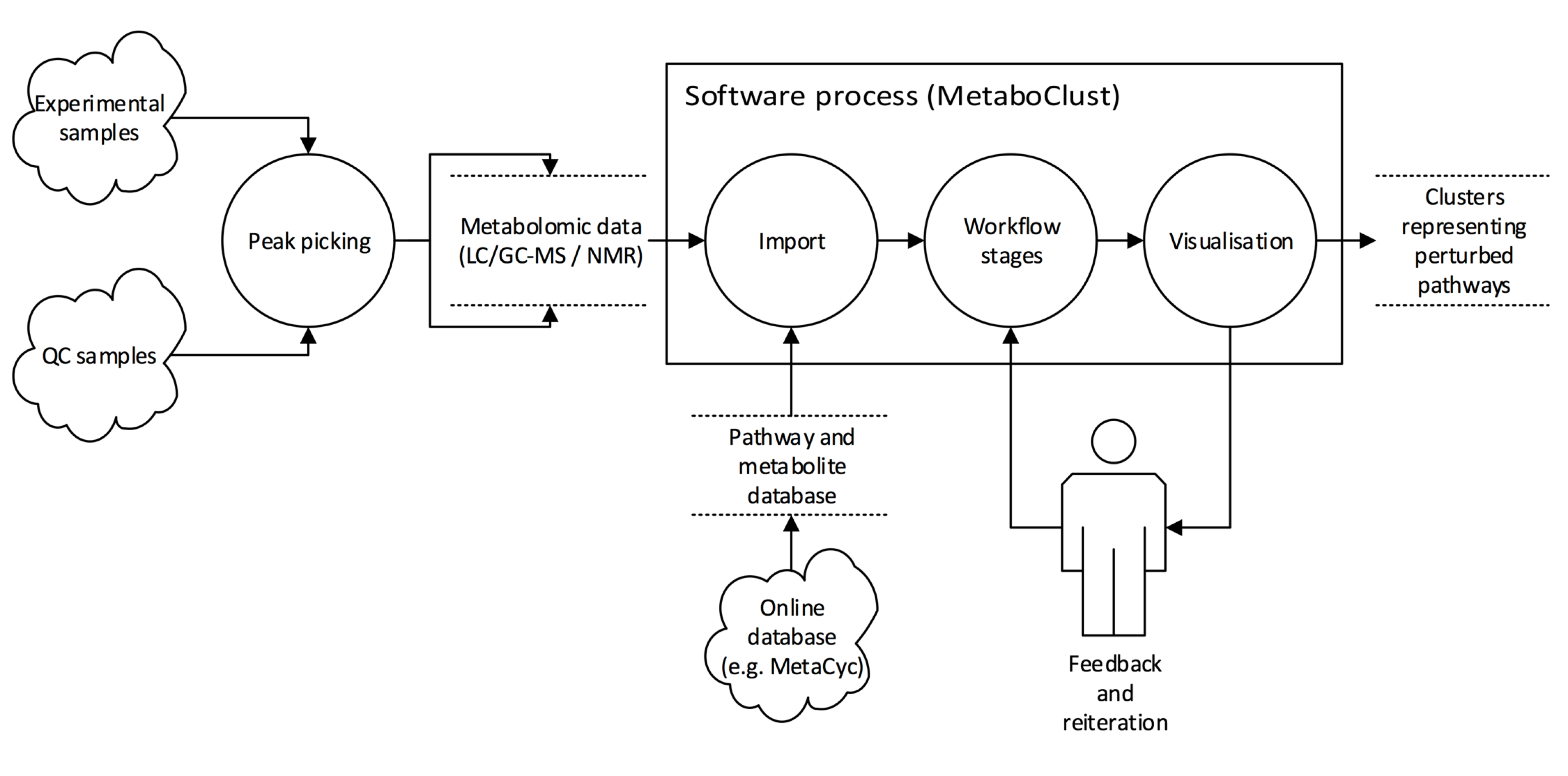

Supplement: S1 Fig — The workflow allows a set of clusters to be generated, representing metabolite concentrations affected by the experimental conditions. Perturbed pathways are suggested by the software, allowing the user to export their data into pathway analysis tools such as MetaCyc (online). Since the workflow is unlikely to be known upfront, the user is actively involved in all stages of analysis. (TIF) [file pone.0205968.s007.tif]

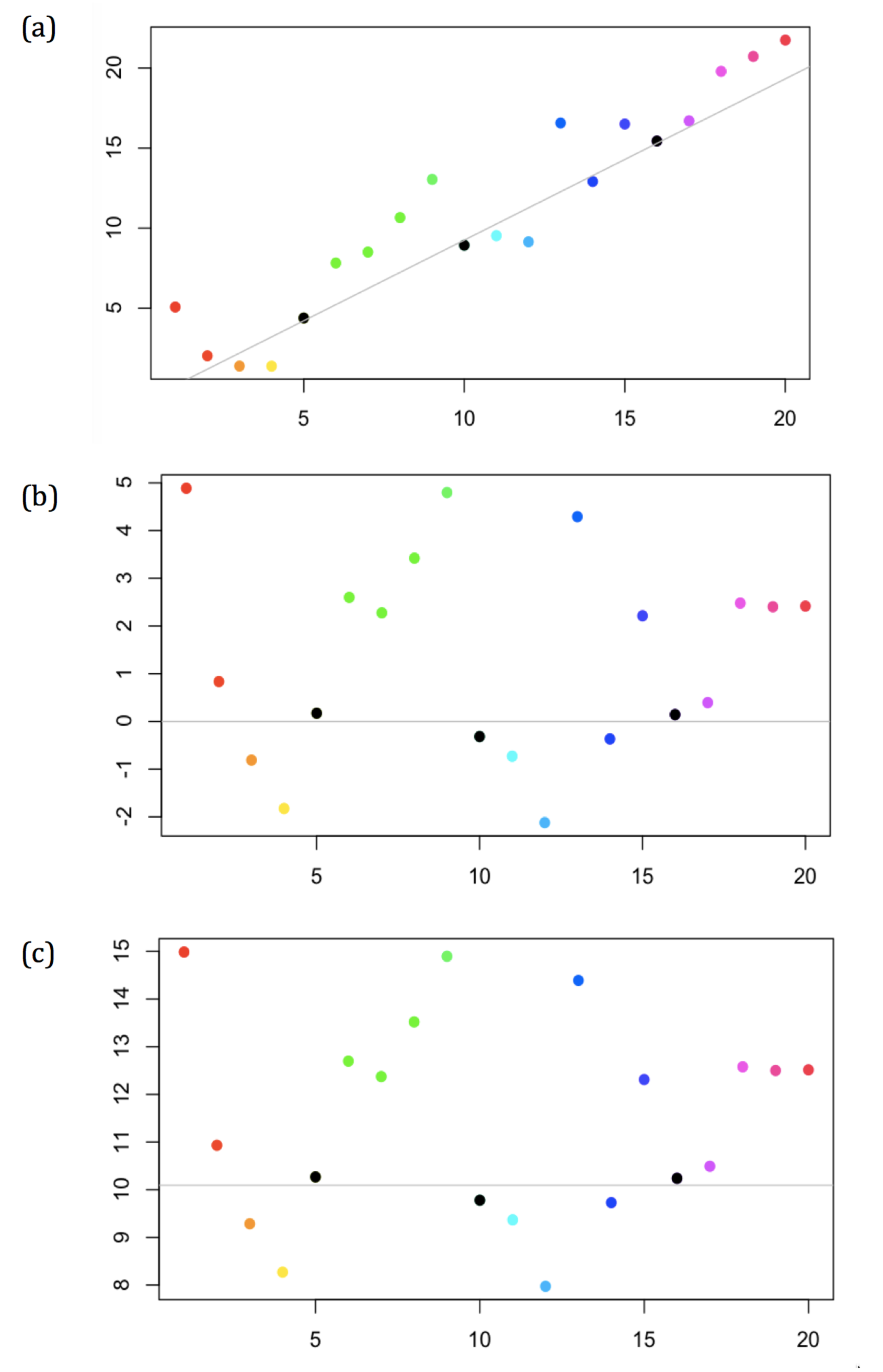

Supplement: S2 Fig — (a) For peak p, the original intensities Xp,b,i for QC samples are indicated by black points and experimental samples are shown in colour. The increasing trend seen in the experimental samples is also evident in the QCs and can be modelled by the regression line obtained from the QCs. The correction factor Cp,b,i in this case is gven by the values predicted by the regression for both QCs and experimental samples. (b) shows the intensities after subtraction of the correction factors with negative values for samples below the regression line. In (c) the horizontal line represents Rp, here taken to be the median value of the original QC intensities, which is then added to obtain the corrected intensities Xp,b,inew=Xp,b,i−Cp,b,i+Rp shown. (TIF) [file pone.0205968.s008.tif]

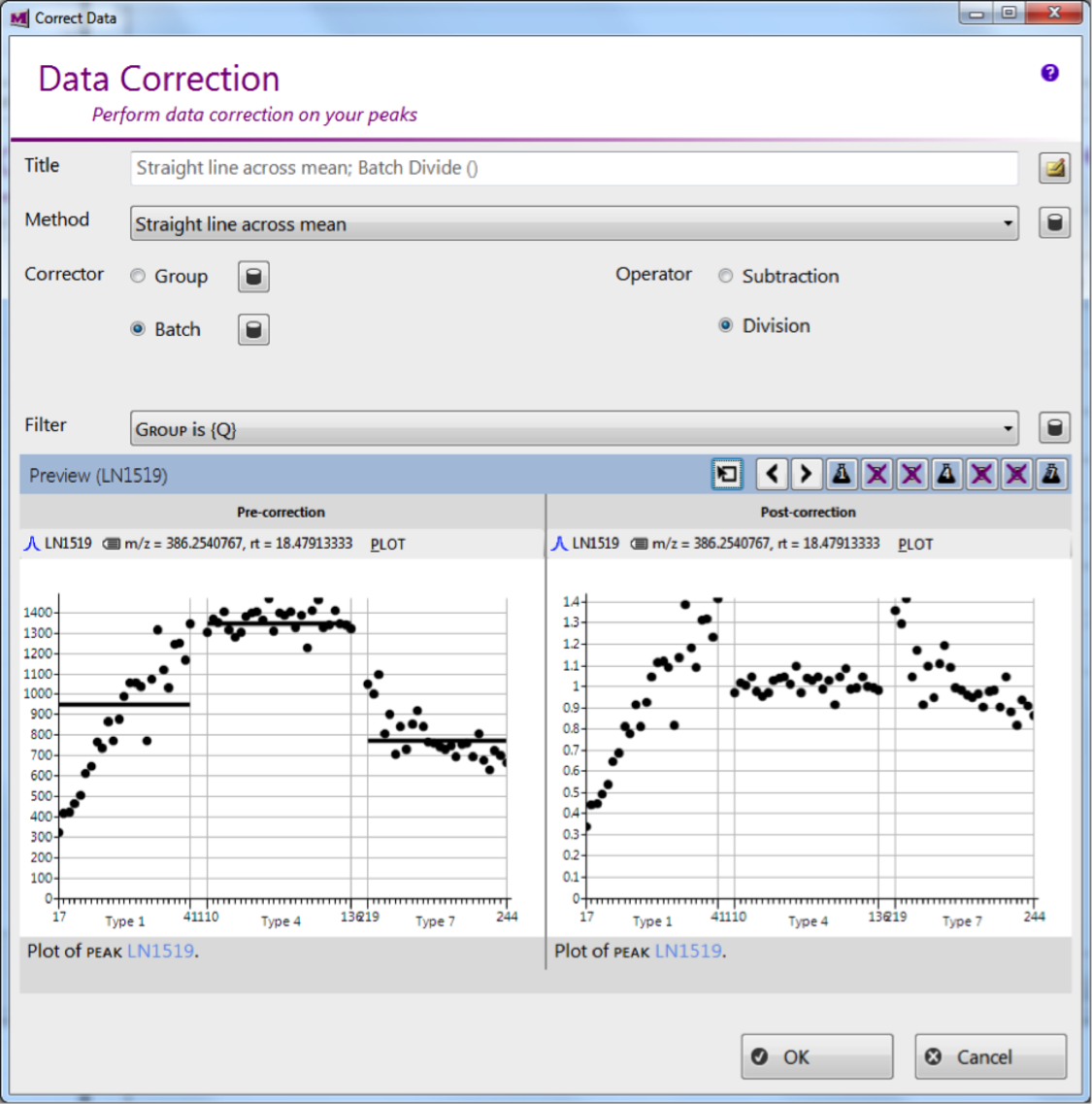

Supplement: S3 Fig — Variations in intensity (Y-axis), both between batches and along the acquisition order (X-axis), can be seen pre-correction (left) as well as post-correction (right) showing that correction is not achieved using this method. (TIF) [file pone.0205968.s009.tif]

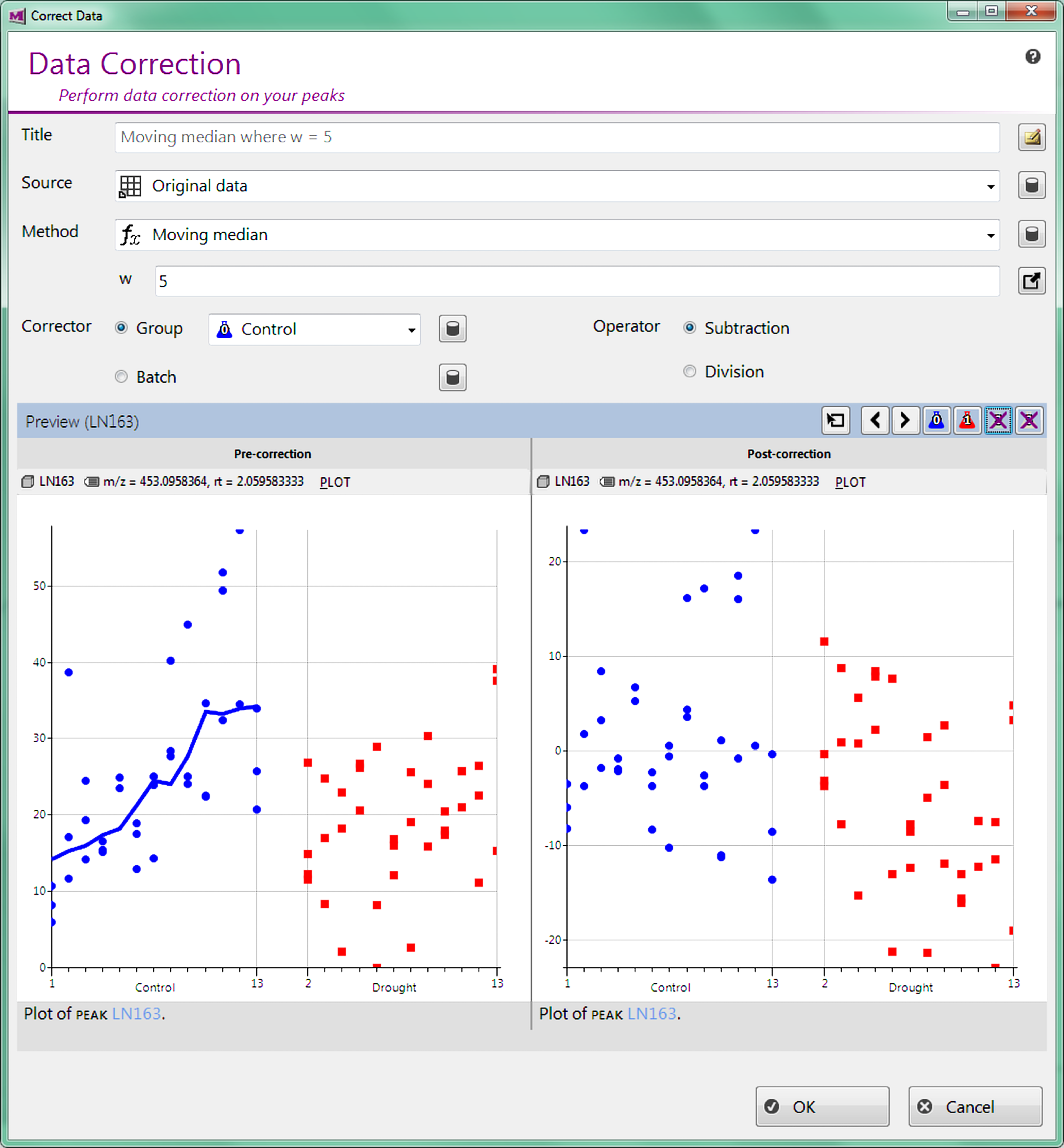

Supplement: S4 Fig — Variations in intensity (Y-axis) due to growth (time, X-axis) can be seen pre-correction (left). Subtraction of the control profile (right) allows the analysis to concentrate on deviations from this profile. Using a smooth trend for the profile avoids the tranfer of noise from the control group into the other experimental groups. (TIF) [file pone.0205968.s010.tif]

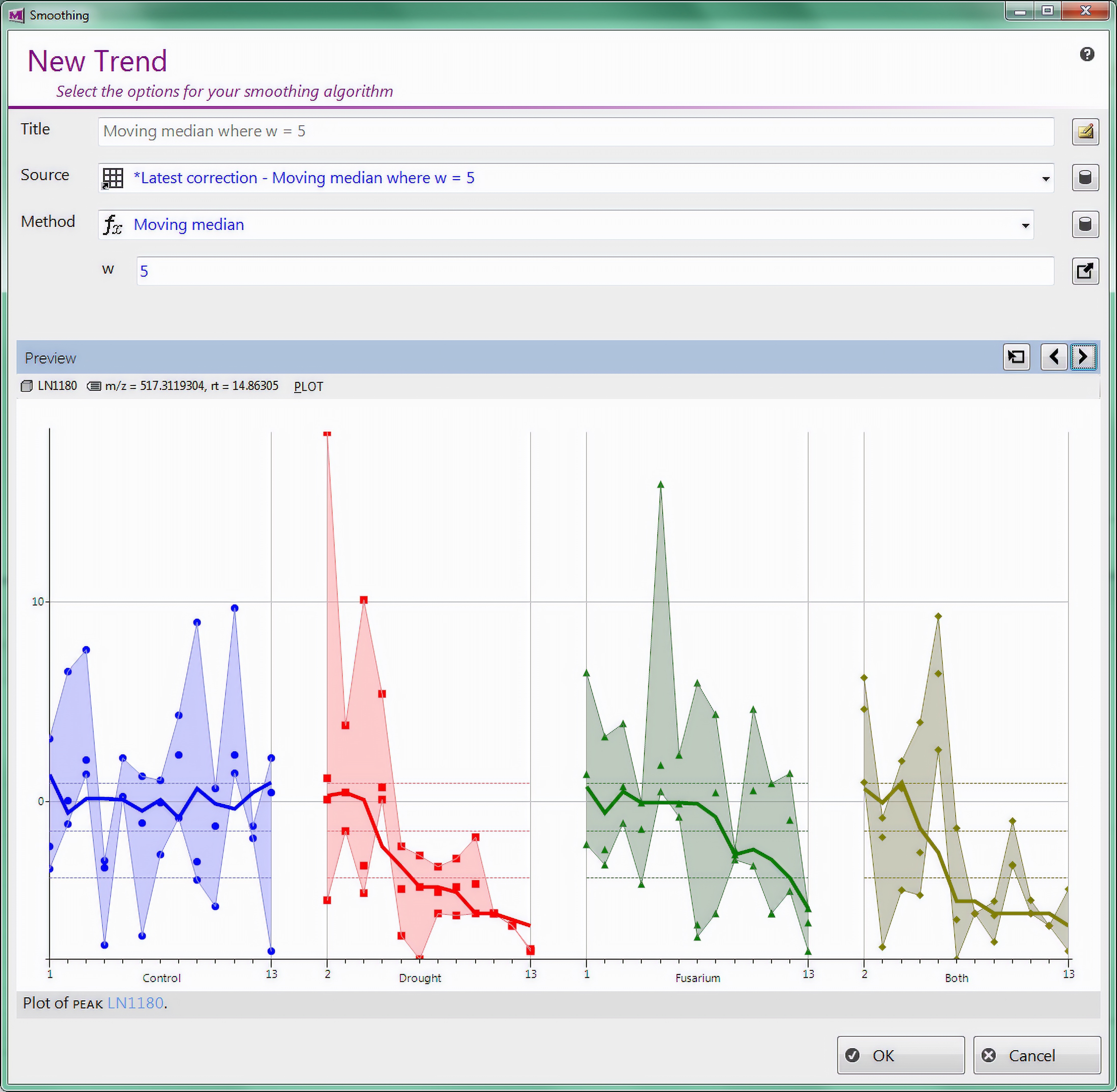

Supplement: S5 Fig — The experimental observations are displayed on the graph, for each experimental group in turn. Blue = control, red = drought stress, green = Fusarium stress, yellow = dual stress. The X axis corresponds to day, and the Y axis to signal intensity. The bold line shows the trend that will be generated for the current settings, in this case a moving median with a window width of 5. (TIF) [file pone.0205968.s011.tif]

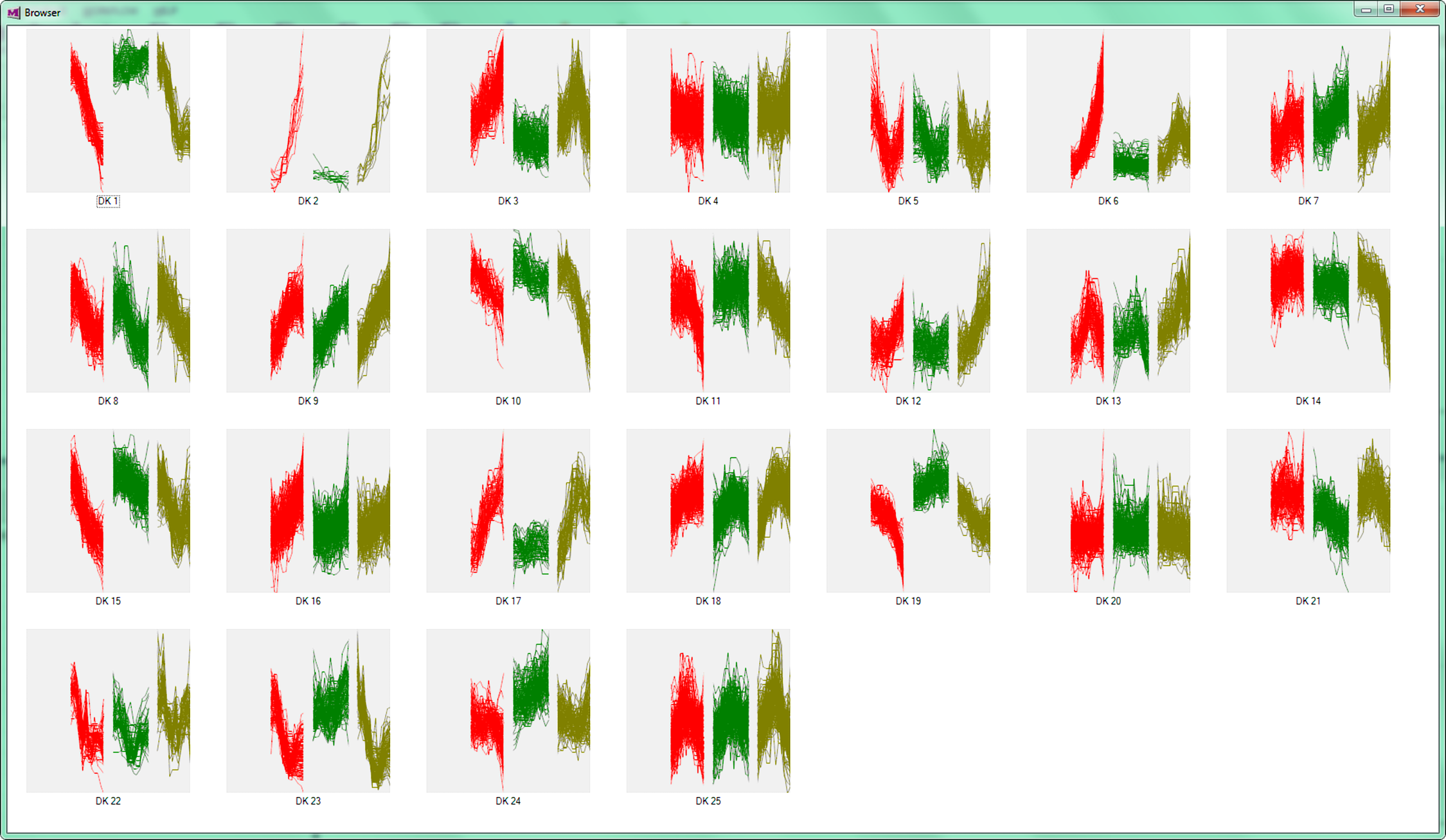

Supplement: S6 Fig — The effects of not filtering the data are apparent in a number of clusters with noisy profiles. Other clusters are also affected by the presence of erratic time profiles. The-X axis corresponds to the input vectors, laid out as drought group, days 2–13 (red); Fusarium group, days 1–13 (green); Dual-stress group, days 2–13 (yellow). The Y-axis corresponds to the auto scaled peak intensity. (TIF) [file pone.0205968.s012.tif]

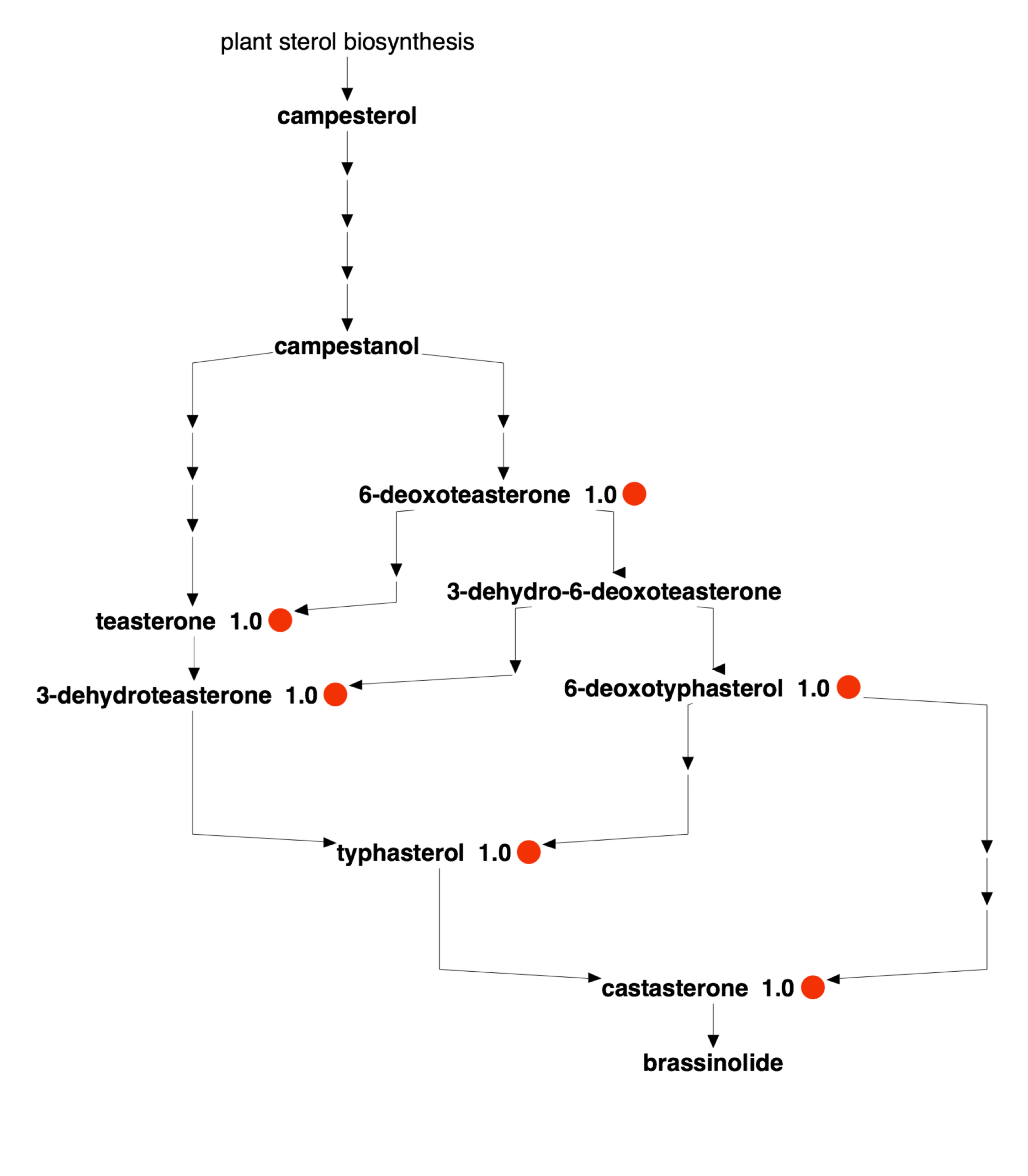

Supplement: S7 Fig — Pathway cluster relationships can be exported from MetaboClust and imported into the MetaCyc online pathway browser. This allows the compounds perturbed by experimental conditions to be highlighted in the pathway. Here the compounds corresponding to annotated peaks in cluster 2 are highlighted by the solid red circles. (TIF) [file pone.0205968.s013.tif]

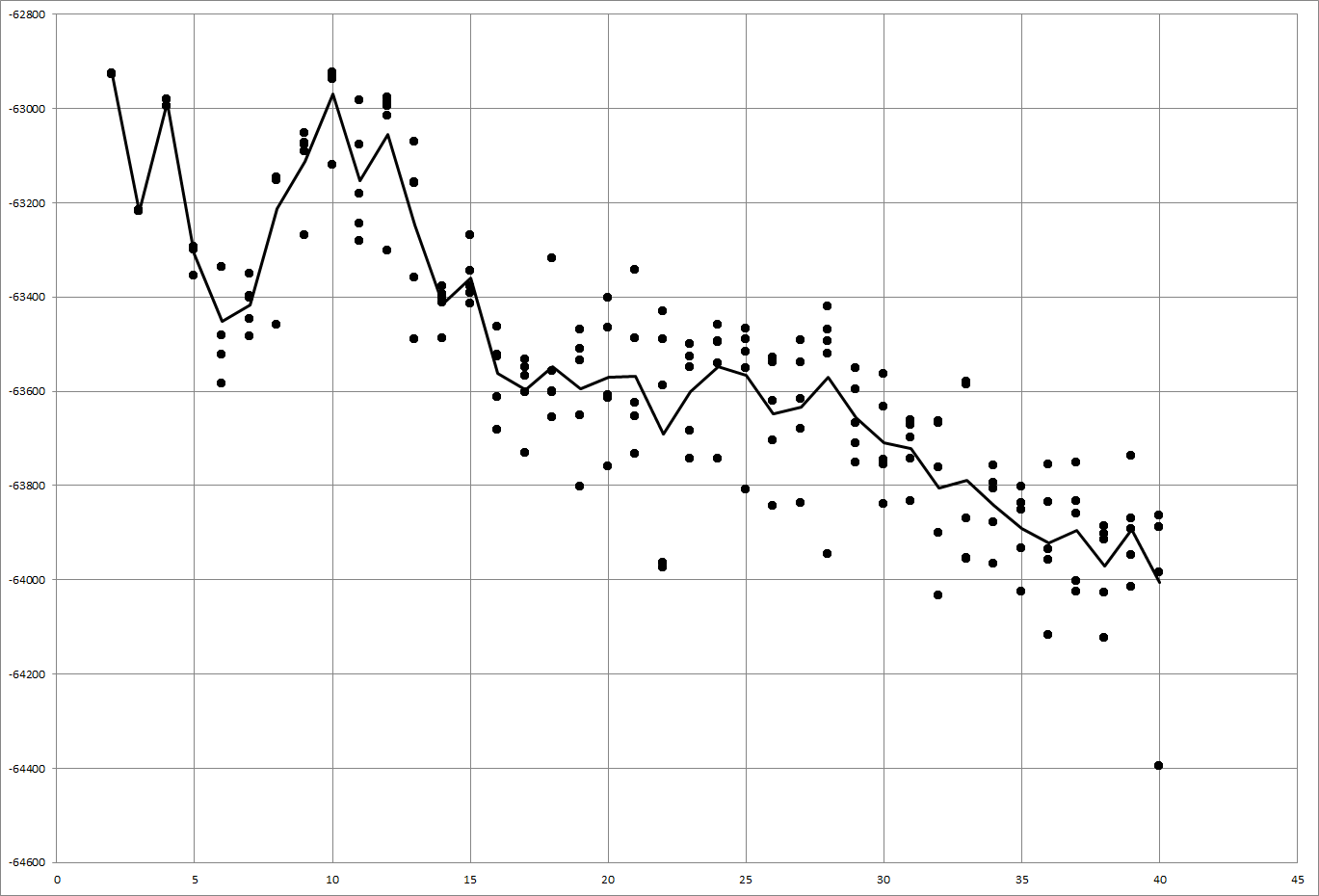

Supplement: S8 Fig — BIC is plotted on the Y axis against the number of clusters, k, on the X axis. Excluding k = 2 the optimal number of clusters is shown to be k = 10. (TIF) [file pone.0205968.s014.tif]
